# Competition between cap and basal actin fiber orientation in cells subjected to contact guidance and cyclic strain

**DOI:** 10.1038/srep08752

**Published:** 2015-03-04

**Authors:** Chiara Tamiello, Carlijn V. C. Bouten, Frank P. T. Baaijens

**Affiliations:** 1 Department of Biomedical Engineering, Eindhoven University of Technology, P.O. Box 513, 5600 MB Eindhoven, The Netherlands; 2 Institute for Complex Molecular Systems, Eindhoven University of Technology, P.O. Box 513, 5600 MB, Eindhoven, The Netherlands

## Abstract

*In vivo*, adhesive cells continuously respond to a complex range of physical cues coming from the surrounding microenvironment by remodeling their cytoskeleton. Topographical and mechanical cues applied separately have been shown to affect the orientation of the actin stress fibers. Here we investigated the combined effects of contact guidance by topographical cues and uniaxial cyclic strain on actin cytoskeleton orientation of vascular derived cells. We devised a modular setup of stretchable circular and elliptic elastomeric microposts, capable to expose the cells to both contact guidance and uniaxial cyclic strain. A competition occurs between these cues when both contact guidance and strain are oriented along the same direction. For the first time we show that this competition originates from the distinct response of perinuclear basal and actin cap fibers: While basal fibers follow the contact guidance cue, actin cap fibers respond to the cyclic strain by strain avoidance. We also show that nuclear orientation follows actin cap fiber orientation, suggesting that actin cap fibers are responsible for cellular reorientation. Taken together, these findings may have broad implications in understanding the response of cells to combined topographical and mechanical cues.

The implication of topographical and mechanical cues in physiological behaviours of adhesive cells such as proliferation, migration and differentiation has become increasingly evident^1^,^2^,^3^,^4^. Consequently, the potential to manipulate cell behaviour by modulating physical cues of the surrounding microenvironment has raised great interest for tissue engineering purposes.

The effects of singular topographical and mechanical cues on the behaviour of adhesive cells have been extensively studied. Cells actively recognize topographical cues ranging from sub-micrometer to ten micrometers in size. As a consequence, cells organize their cytoskeleton in order to align and migrate along the topographical cue. This phenomenon is known as contact guidance^5^,^6^,^7^,^8^. Furthermore, the impact of mechanical loading on cellular behaviour has also been of broad attention. For instance, epithelial cells, endothelial cells and adjacent fibroblasts withstand and respond to strains in the surrounding microenvironment resulting from physiological processes such as embryonic development^9^, blood pulsation^10^,^11^ and muscular contraction respectively^12^. *In vitro* investigations revealed that uniaxial cyclic strain has an effect on the alignment of cells. Most types of adhesive cells respond to uniaxial cyclic strain with a strain avoidance response, thus by reorienting almost perpendicular to the strain direction^13^,^14^,^15^,^16^. Taken together, the results of these studies suggest that topographical and mechanical cues might act as competitive cues for cellular alignment. However, investigations and understanding of the influence and interplay of topographical and mechanical cues on cell behaviour are currently lacking. This kind of studies would give insight on cell adaptation in the native cellular microenvironment, which includes a combination of topographical as well as mechanical cues. Furthermore, findings from these studies would be of great benefit for the area of (in-situ) tissue engineering of load-bearing tissues. In micro structural scaffolds, cell organization and subsequent neo-tissue formation are indeed a function of both topographical cues and (cyclic) strain imposed on the tissue.

The ability of adhesive cells to sense and respond to the physical properties of their microenvironment lies in the interaction and communication between the cells and the surrounding environment, which results in the translation of physical stimuli into biochemical signals (mechanotransduction)^17^. Key cellular components for mechanotransduction are focal adhesions and actin stress fibers. These elements are responsible for cellular mechanosensing and propagation of the signals to the nucleus. By the transduction of the signals to the genome, the final adaption of the cell to the developing microenvironment occurs^18^. However, recent studies report on the existence of a direction transduction mechanism going from the surrounding mechanical stimuli of the extracellular environment to the nucleus^19^. This physical interconnection is the perinuclear actin cap, a subset of actin stress fibers running on top of the nucleus and directly interacting with it via nuclear membrane proteins. The perinuclear actin cap has been shown to dominate the (early) mechanoresponse of adherent cells, as opposed to basal fibers which do not have a direct connection to the nucleus^19^,^20^,^21^,^22^,^23^.

In this study, we concentrate on the combined effects of topographical and mechanical cues, namely contact guidance and uniaxial cyclic strain, on the actin cytoskeleton of vascular derived cells (HVSC). In particular, we aim at understanding which cue dominates the response of stress fiber orientation, when uniaxial cyclic strain and contact guidance are applied together. We cultured HVSC on top of a modular setup of arrays of elastomeric microposts of different lengths (1, 3 and 6 μm), cross sections (circular and elliptical) and bound to a stretchable membrane. By analyzing stress fiber orientation, we were able to dissect between the effects of stiffness, contact guidance and cyclic strain. We observed that stress fiber orientation does not depend on the stiffness. Instead, stress fiber orientation is correlated with the presence of contact guidance and the relative angle between it and the uniaxial cyclic strain. We demonstrate that a competition between contact guidance and strain arises when both cues were applied along the same direction. Further examination of the actin architecture pointed out that such effect involves the distinct response of the perinuclear actin cap fibers and the basal stress fibers. While actin cap fibers are prone to a strain avoidance response, basal actin fiber orientation remains dominated by the contact guidance of the underlying microposts.

## Results

### Modular setup

The developed modular setup for investigating the combined effects of contact guidance, stiffness and strain on stress fiber orientation consisted of microfabricated elastomeric micropost arrays characterized by different cross sections and lengths, incorporated into a stretching device.

**Figure 1:**
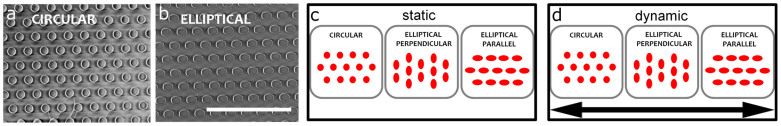
Modular setup of elastomeric micropost arrays and experimental conditions. Representative scanning electron micrographs (top view) of hexagonally arranged elastomeric microposts forming a dense hexagonal lattice. Both circular cross section microposts (circular, a) and elliptic cross section microposts (elliptical, b) are shown. Scale bar: 20 μm. Overview of static (c) and dynamic (d) experimental conditions. Schematic representation of neighboring arrays of elastomeric microposts representing the three experimental conditions in red. 1) Circular microposts, 2) elliptical perpendicular microposts characterized by elliptic cross section which major axis is perpendicular to the strain direction and 3) elliptical parallel microposts characterized by elliptic cross section which major axis is parallel to the strain direction. The double-arrow headed line represents the strain direction for the dynamic condition.

By changing the cross sections we fabricated anisotropic and isotropic microposts, which respectively do and do not induce cellular contact guidance. The geometrical anisotropy was given by an elliptic cross section, which directed cell alignment along the major axis of the ellipse. On the other hand, random cell alignment was obtained with isotropic microposts, which were characterized by a circular cross section (Fig. 1a).

The choice of three microposts lengths (1, 3 and 6 μm) allowed mimicking different substrate stiffnesses (Table 1). In fact, the bending stiffness of each micropost depends on its length when the cross section is kept unchanged^47^,^48^. Finally, the micropost arrays were designed in array clusters that represent different experimental conditions. As a result, we could study the response to several topographical conditions in one experiment. The experimental conditions studied are referred to as circular, elliptical perpendicular and elliptical parallel, where perpendicular (parallel) means that the major axis of the elliptic cross section is perpendicular (parallel) to the strain direction (Fig. 1b).

For control studies in static conditions (static), the micropost arrays were bound to glass coverslips. Instead, for studying the effects of uniaxial cyclic strain (dynamic), the arrays were bound to commercially available flexible-bottomed culture wells. The uniaxial cyclic strain applied to the membrane bound to the microposts was transferred to the micropost tops and, therefore, was able to exert strains to the adherent cells attached to the micropost tops^24^.

### Contact guidance dictates stress fiber orientation independent of micropost stiffness under static conditions

**Figure 2:**
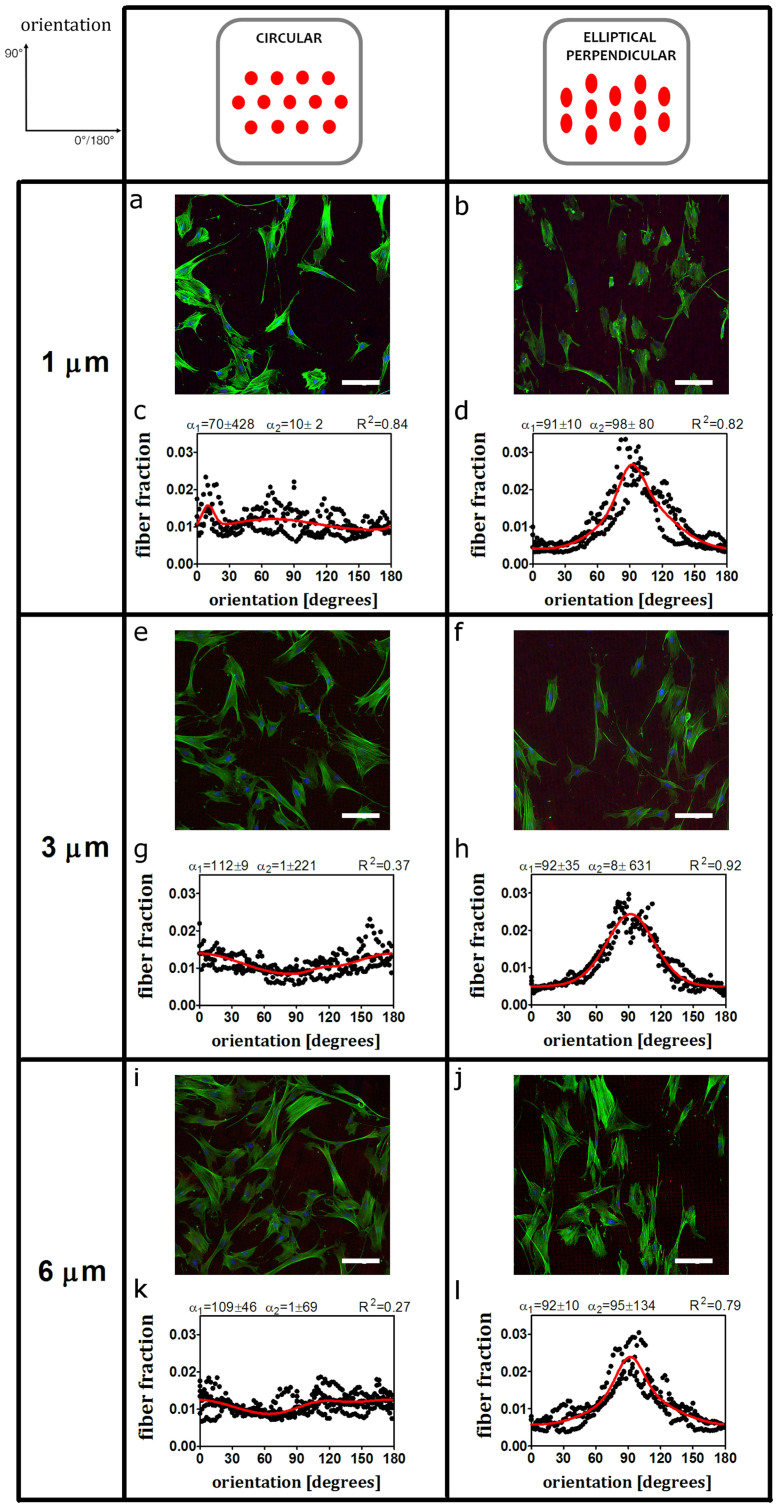
Stress fiber orientation follows contact guidance prescribed by the major axis of elliptical microposts while it is random on circular microposts. Representative fluorescent images (actin stress fibers in green, nucleus in blue, rhodamine conjugated fibronectin coated on top of microposts in red) and bimodal fits of the stress fiber orientation (including the first and the second dominant fiber angle with standard deviations and R-squared value). (a, c, e, g, i and m) For circular microposts stress fibers do not show a preferred orientation. (b, d, f, h, l and n) For elliptical perpendicular, stress fibers align with the micropost major axis (about 90°). Scale bars represent 100 μm. The data reported are results from n = 3 independent experiments, at least 40 cells were considered per each condition. Results for elliptical horizontal microposts were omitted.

To validate our modular setup, HVSC were cultured in static conditions on circular and elliptical microposts coated with rhodamine labelled fibronectin. After 33 hours cells were fixed and observed for stress fiber orientation. While no preferred orientation was observed on circular microposts (Fig. 2 a, c, e, g, i and k) stress fibers aligned with the major axis of the elliptical perpendicular microposts (Fig. 2 b, d, f, h, j and l).

Examination of stress fiber orientations on micropost lengths of 1, 3 and 6 μm, respectively, showed similar stress fiber distributions for the circular and elliptical perpendicular cases indicating that the stress fiber distributions are independent of the micropost bending stiffness (Fig. 2 and Supplementary Fig. S1 online).

### Competition between contact guidance and strain avoidance determines stress fiber orientation

**Figure 3:**
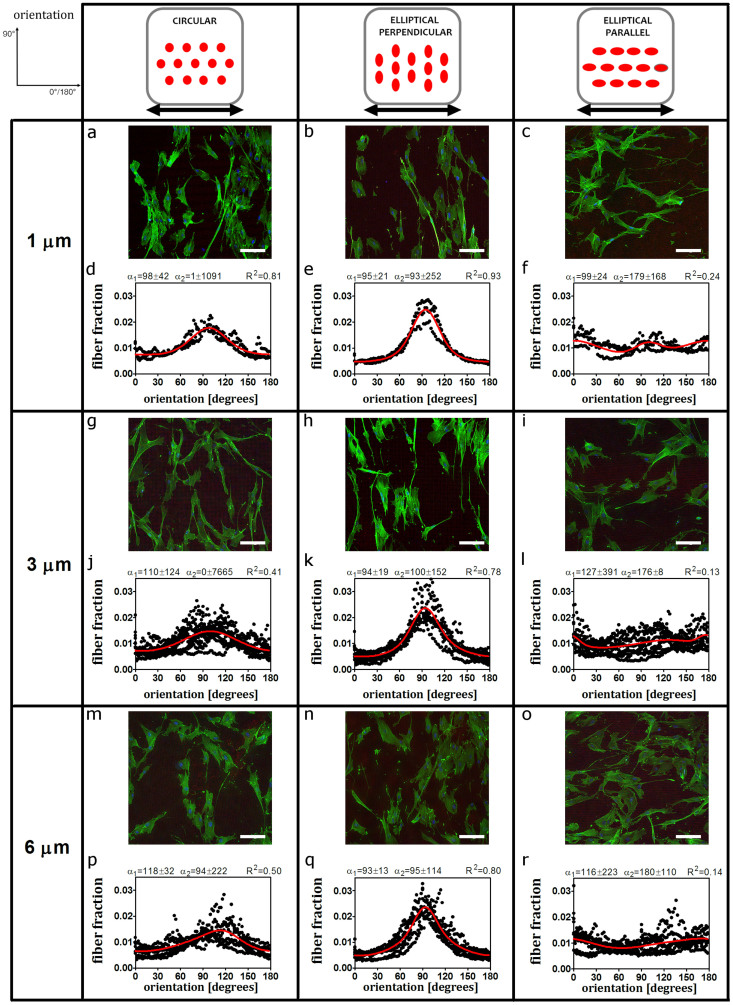
Stress fiber orientation shows strain avoidance response on circular and elliptical perpendicular microposts, but not on elliptical parallel microposts after uniaxial cyclic strain. Outcomes of stress fiber orientation upon uniaxial cyclic strain for circular, elliptical perpendicular and elliptical parallel microposts. Representative fluorescent images (actin stress fibers in green, nucleus in blue, rhodamine conjugated fibronectin coated on top of microposts in red) and bimodal fits of the stress fiber orientation (including the first and the second dominant fiber angle with standard deviations and R-squared value) after 19 hours of uniaxial cyclic strain prescribed in the horizontal direction corresponding to a 0°/180° angle. (a, d, g, l, o and r) For circular microposts subjected to dynamic loading, stress fibers reorient shows strain avoidance (perpendicular to the strain direction). (b, e, h, m, p and s) For elliptical perpendicular, stress fibers align with the micropost major axis which is also perpendicular to the mechanical load. (c, f, i, n, q and t) For elliptical parallel microposts, no preferred stress fiber alignment was observed. Scale bars represent 100 μm. The data reported come from n = 3 independent experiments in case of 1 μm microposts, n = 7 independent experiments in the other cases.

To study the relative impact of contact guidance and uniaxial cyclic straining on stress fiber orientation, we exposed HVSC seeded onto elastomeric microposts to uniaxial cyclic straining in the horizontal direction at 0.5 Hz in frequency and 6.8% in amplitude for 19 hours. The stress fiber distribution was analysed after cell fixation (Fig. 3). On circular microposts, stress fibers displayed a strain avoidance response, as they oriented toward the (near) perpendicular alignment with respect to the strain direction (Fig. 3 a, d, g, j, m and p). On elliptical perpendicular microposts, in dynamic conditions, stress fibers exhibited alignment with the major axis of the elliptical microposts (Fig. 3 b, e, h, k, n and q). This result was comparable to the stress fiber orientation in static conditions and shows that stress fibers remained perpendicular to the strain direction. In contrast, on elliptical parallel microposts, we observed a change in the stress fiber distributions compared to the static conditions. The lack of a preferred orientation pointed to a competition between strain avoidance and contact guidance due the uniaxial cyclic straining and contact guidance invoked by the elliptic cross section of the microposts (Fig. 3 c, f, i, l, o and r).

In summary, these results show that stress fiber reorientation is affected by the orientation of the elliptical microposts compared to the straining direction. Indeed, on the elliptical parallel microposts the application of strain results in hindered strain avoidance when compared to circular microposts.

In agreement with our findings under static conditions, stress fiber orientation under uniaxial cyclic straining did not show any correlation with the bending stiffness of the (Fig. 3 and Supplementary Fig. S1 online).

### Distinct responses of basal and perinuclear actin cap fibers to contact guidance and cyclic strain explain the competition between these cues

**Figure 4:**
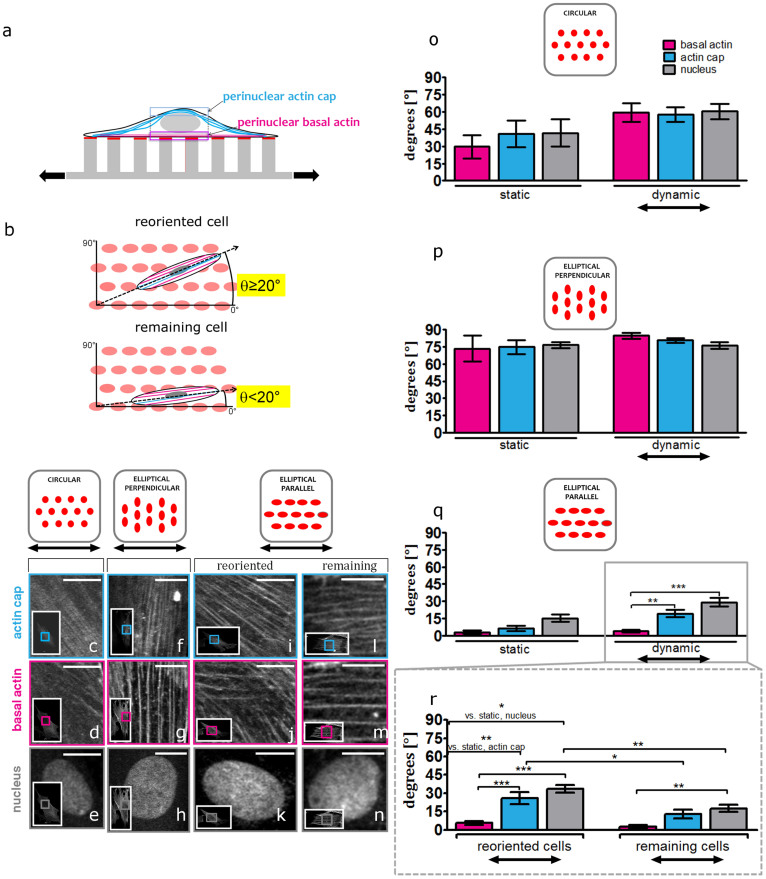
Basal stress fibers follow contact guidance, while actin cap stress fibers and nucleus display strain avoidance. (a) Schematic of an adhesive cell cultured on top of stretchable microposts coated with rhodamine conjugated fibronectin (red). Status of actin organization at the perinuclear actin cap (light blue) and basal (magenta) is examined by confocal microscopy. (b) Schematic representation of the classification of reoriented (top) and remaining (bottom) cells on top the elliptical parallel microposts. The orientation angle θ between the micropost major axis and the major axis of a cell determined by fitting an ellipse to the cell's outer boundary. If θ ≥ 20°, the cell is categorized as reoriented, while, if θ<20°, the cell is categorized as remaining. (c–n) Representative confocal fluorescent micrographs of perinuclear actin cap fibers, basal fibers and nucleus of HVSC cultured on circular, elliptical perpendicular and elliptical parallel microposts (reoriented (left column) and remaining cell (right column)) after 19 hours of uniaxial cyclic strain applied in the horizontal direction corresponding to a 0° angle. Insets show the whole imaged cell, with inner boxes framing the zoomed regions shown in the main panels. For all images in this figure actin stress fibers are visualized with FITC-Phalloidin. Scale bars represent 10 μm (o–q) Mean orientation of basal and actin cap fibers and nucleus in static conditions (left side) and after 19 hours of uniaxial cyclic strain (dynamic) (right side) for circular (o), elliptical perpendicular (p) and elliptical parallel microposts (q). In panel o, 5 cells in static and 12 cells in dynamic conditions were analyzed. In panel p, 6 cells in static and 14 cells in dynamic conditions were analyzed. In panel q, 6 cells in static and 24 cells in dynamic conditions were analyzed. (r) Mean orientation of basal and actin cap fibers and nucleus in dynamic conditions for reoriented (n = 13) and remaining (n = 11) cells on elliptical parallel microposts. For all graphs, values represent means ± SEM. ***, **, * indicate p value <0.001, <0.01, <0.05 respectively.

We investigated whether the stress fiber organization within the cell itself can explain the results of the combined effects of contact guidance and uniaxial cyclic strain. Therefore, we acquired z-stacks of confocal images of actin fibers stained of HVSC cultured on 1 μm micropost arrays in static and dynamic conditions. We subsequently quantified the mean orientation of the perinuclear stress fibers both at the basal and actin cap levels (Fig. 4a).

In all static experimental conditions, the preferred orientation of basal and actin cap fibers was similar (Fig. 4 o, p and q (static)) indicating that in both zones of the cell, actin fibers ran invariably parallel to each other in HVSC adhered to circular and elliptical microposts. However, under dynamic conditions, the combination of uniaxial cyclic strain and contact guidance had distinct effects on basal and actin cap fiber orientation depending on the experimental condition. On circular microposts, uniaxial cyclic strain elicited reorientation of both basal and actin cap fibers to a mean orientation of <α> ≈ 59°. The shift between the mean orientation at the basal and actin cap levels was not significant (<α> ≈ 60° and <α> ≈ 59° for basal and actin cap respectively), indicating that both basal and actin cap fibers showed a similar strain avoidance response (Fig. 4 c, d and o). In case of elliptical perpendicular microposts the mean orientations of basal and actin cap fibers were not significantly different compared to the static condition (Fig. 4p). The mean direction was <α> ≈ 85° and <α> ≈ 81° for basal and actin cap fibers respectively. This indicates that stress fibers remained almost perpendicular to the strain direction, which is also the direction of the contact guidance provided by the microposts (Fig. 4 f and g). In contrast, on elliptical parallel microposts we measured a significant shift between the mean orientation of basal and actin cap fibers. While the basal fibers had a mean orientation of <α> ≈ 4° and thus were mainly oriented along the micropost major axis, the actin cap fiber mean orientation shifted to <α> ≈ 19° (**p<0.01), reflecting the tendency of the basal fibers to remain aligned with the micropost major axis, thus with the contact guidance (Fig. 4q). Instead, actin cap fibers were sensitive to the uniaxial cyclic strain cue and, consequently, they tended to orient away from the strain direction, neglecting the contact guidance cue.

We also assessed cell orientation in HVSC adhered to elliptical parallel microposts in dynamic conditions, as we observed that about half of the cells remained parallel to the microposts while about half reoriented in a different direction. We classified as “reoriented” the cells which orientation angle was θ>20° (relative to the micropost major axis and strain direction) and as “remaining” the ones which orientation angle was θ<20° (Fig. 4b). In reoriented cells the difference between actin cap and basal fibers was ≈ 20° (<α> ≈ 6° for basal and <α> ≈ 26° for actin cap, ***p<0.001) (Fig. 4 i, l and t (left). Instead, for the remaining cells, the shift was not significant ≈10° (<α> ≈ 3° for basal and <α> ≈ 13° for actin cap) (Fig 4 l, m and r (right)). Summarizing, these data demonstrate a zone-based response to contact guidance and uniaxial cyclic straining due to the basal fibers and actin cap fibers independently.

Z-stacks were also studied to determine the impact of contact guidance and uniaxial cyclic straining on nuclear orientation. Since the actin cap is physically connected to the nucleus, we hypothesized that the actin cap orientation would mediate nuclear reorientation^20^. To test this hypothesis we measured the nuclear orientation and compared it with the mean actin cap fiber orientation. Under static conditions, nuclei were oriented in the direction of actin cap fibers, which ran parallel to the basal fibers (Fig. 4 o, p and q (static)). Upon uniaxial cyclic straining, the nucleus was oriented in the same direction as actin cap fibers in all the experimental conditions (Fig. 4 e, h, k, n, o, p, q and r). These results indicate that a correlation exists between nuclear and actin cap orientation.

## Discussion

The main objective of this investigation was to gain insight on the stress fiber orientation in vascular derived cells subjected to contact guidance and uniaxial cyclic strain. To do this we devised a modular setup made of stretchable elastomeric microposts capable to invoke cellular contact guidance. Analysis of stress fiber orientation at the cell level revealed that neither contact guidance nor strain avoidance dominates when contact guidance and strain are prescribed along the same direction. Our findings at actin fiber level give further explanation for this observation: Two distinct responses are observed in perinuclear basal and actin cap fibers. Within the experimental timeframe (19 hours) and the strain regime considered, perinuclear actin cap fibers predominantly respond to uniaxial cyclic strain and not to contact guidance, while basal stress fiber orientation is dictated by contact guidance only.

Our study also shows that the modular setup of stretchable elastomeric microposts can be used to study the independent and combined effects of contact guidance and strain on stress fiber orientation of adhesive cells. Since in static conditions we observed stress fiber alignment along the major axis of the elliptical microposts, we show evidence that our system can impose contact guidance.

We were also able to simulate a range of substrate stiffnesses, by changing the micropost length (Table 1). However, we did not detect any difference in stress fiber orientation, in both static and dynamic conditions. Presumably, in the range of the stiffnesses studied, the arrangement of stress fibers, which correlates with the arrangement and maturation of focal adhesions, could be regulated by the topographical cues only and not by stiffness. This observation is consistent with the results of Seo *et al.*, who reported regulation of focal adhesions localization by topographical variations in micro patterns, independently of the substrate stiffness^25^. Yet, the cellular stiffness sensing mechanism is a subject of intensive debate because conflicting evidences have emerged from recent studies trying to elucidate whether bulk substrate stiffness or extracellular matrix protein tethering regulates the mechanosensitive cellular response^26^,^27^,^28^,^29^,^30^,^31^.

Our observations about stress fiber reorientation on circular microposts are consistent with studies on flat, isotropic, 2D substrates subjected to cyclic uniaxial strain^14^,^15^,^16^,^32^,^33^. We show indeed that stress fiber orientation follows a strain avoidance response. The effects on cellular orientation upon simultaneous stimulation by contact guidance and mechanical loading was topic of previous studies. However, in these investigations, micro-structures such as grooves^33^,^34^,^35^ or micro-patterned lines^36^,^37^ were employed. The spatial confinement of focal adhesions and stress fibers in the z-direction resulting from the use of grooves could be critical for stress fiber reorientation^38^. In case of micro-patterned lines used to impose cell alignment, stress fiber reorientation is restricted to the width of the lines. To avoid these concerns, and in order to allow free cell and stress fiber reorientation, we have used contact guidance invoked by elliptical micropost cross sections. The perpendicular and parallel arrangement of the elliptical microposts enabled us to show the direct effects on stress fiber reorientation of uniaxial cyclic strain combined with contact guidance. We demonstrated that the combination of the two cues does not affect stress fiber reorientation in case of elliptical microposts oriented perpendicular to the strain direction. In this case, both basal and actin cap fibers remained oriented perpendicularly to the strain direction. Thus, it is likely that cells grown perpendicularly to the strain direction become insensitive to strain, as they can maintain their orientation^39^. Conversely, uniaxial cyclic strain and contact guidance resulted in antagonistic responses when we exposed the cells to both cues applied along the same direction. In this case the strain avoidance response was only partly seen; about half of the cells remain aligned with the micropost and half of the cells reorient away from the strain direction. When looking at the whole group of cells, we found that the perinuclear actin cap fibers reoriented at about 19° to the strain/micropost major axis direction, while the perinuclear basal fibers remained aligned with the underlying microposts. This observation can probably be attributed to two reasons. Firstly, the enhanced mechanosensing characterizing the actin cap associated focal adhesions make them more sensitized for mechanical loading. Actin cap associated focal adhesions have indeed been shown to differ from basal actin fiber associated conventional focal adhesions, e.g. they dominate cell mechanosensing over a wide range of matrix stiffness^20^. Secondly, each actin cap fiber is anchored to the microposts only at the two actin cap associated focal adhesions. This makes the actin cap less entangled to the underlying microposts as compared to the basal stress fibers, which instead contact the microposts in numerous conventional focal adhesions. As a consequence, the response of actin cap fibers mechanical loading can be more dynamic. Being both explanations not mutually exclusive, we suggest that the peculiarity of the actin cap focal adhesions and the structural organization of the actin cap itself might both play a role in triggering the strain avoidance response of actin cap fibers.

Additionally, we revealed a correlation between actin cap fiber orientation and nuclear orientation both in static and dynamic conditions. In particular, even when uniaxial cyclic strain was applied along the same direction of contact guidance, we observed that actin cap fibers and nucleus oriented at the same angle. These observations lead us to hypothesize that the reorientation of the actin cap brings along nuclear reorientation. We relate this phenomenon to the fact that the actin cap is directly anchored to the nucleus^23^ through linkers of nucleoskeleton and cytoskeleton complexes^40^. In support to this, a recent study by Kim *et al.* also demonstrates the direct involvement of actin cap fibers in controlling nuclear rotation and translocation^41^.

**Figure 5:**
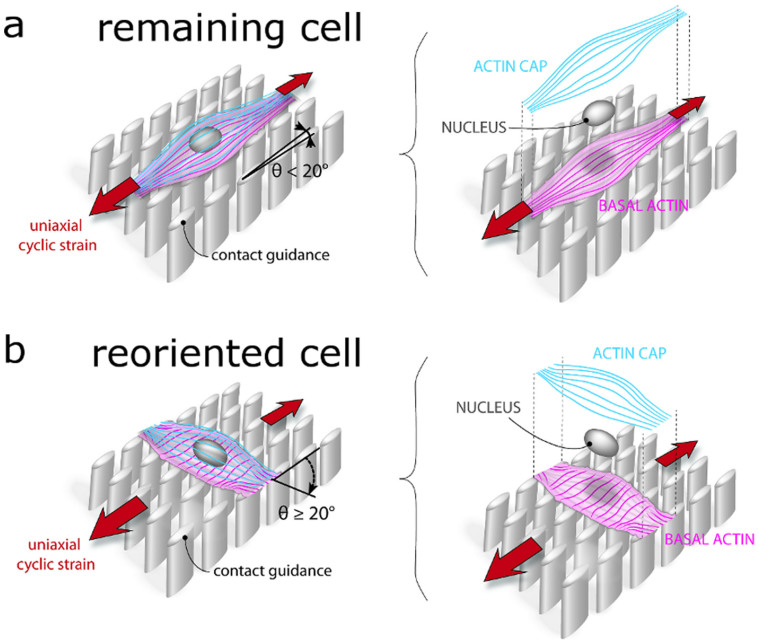
Schematic drawing of the effects of uniaxial cyclic strain on actin stress fiber orientation of vascular derived cells cultured on elliptical microposts aligned with the direction of strain. Our results revealed two kinds of cellular responses: Remaining and reoriented cells. (a) The remaining cells remain aligned with the contact guidance cue provided by the micropost elliptical cross section. The perinuclear basal and actin cap fibers run parallel to each other. (b) Reoriented cells orient themselves at an angle ≥20°. This orientation is mainly given by actin cap fibers orientation. These fibers have a strain avoidance response. Perinuclear basal actin fibers instead do not reorient but remain aligned with the contact guidance cue. The nuclear orientation is similar to the actin cap orientation. Figure by Anthal Smits.

Taken all the results into consideration, within the experimental conditions of this study, we speculate the following mechanistic model for vascular derived cells grown on elastomeric microposts exposed to uniaxial cyclic strain. In absence of contact guidance, actin cap fibers respond to uniaxial cyclic strain by reorienting towards a direction almost perpendicular to the strain. Therewith, the physical anchorage of the actin cap to the nucleus results in the reorientation of the nucleus co-aligned with actin cap fibers. While being reoriented, the nucleus and, thus, the genome receive the signals coming from the actin cap mechanosensing. This leads the nucleus to orchestrate the reorientation of the whole cell, including the reorientation of basal stress fibers, and thus the reorientation of the whole cell. If contact guidance and uniaxial cyclic strain are applied in the same direction, an active competition between the cues arises resulting in half cell population remaining aligned with the contact guidance cue and half reorienting at an angle ≥20° from the contact guidance cue (Fig. 5). Noticeably, in reoriented cells, actin cap fibers and the nucleus reorient at an angle to the strain direction, while the perinuclear basal actin fibers remain aligned with the contact guidance cue.

Clearly it needs to be further investigated whether such cell behaviour is dependent on the straining regime and the experimental time frame. Moreover, it will be important to consider the influence of cell density, as the formation of a confluent cell sheet might alter the actin cap fiber behaviour.

In conclusion, this study sheds light on the response of the actin cytoskeleton and the nucleus of vascular derived cells subjected to a combination of topographical and mechanical cues such as contact guidance and uniaxial cyclic strain on planar substrates. We show that the combination of these cues can result in competing effects on the stress fiber orientation response. We demonstrated that actin cap fibers have a pronounced response to strain and are responsible for nuclear strain avoidance response. On the other hand, the perinuclear basal actin fibers appear to be more sensitized to the contact guidance cue. These findings have implications for tissue engineering where contact guidance and uniaxial cyclic strain are involved.

## Methods

### Cell cultures

The cells used in this study are human vascular-derived cells (HVSC) harvested from the vena saphena magna from the Catharina Hospital Eindhoven. The tissues are considered surgical rest material, whereby the patient has been informed on potential use of rest material for scientific research purposes^42^. Verbal informed consent was obtained from patients and tissues were handed over anonymously, without any patient-specific information except for gender. Procedures for secondary use of patient material were followed as described in the Dutch code of conduct for responsible use of patient material. According to the Dutch medical scientific research with human subjects act (WMO), secondary use of patient material does not need review by a Medical Ethics Examination Committee. HVSC have previously been characterized as myofibroblasts^43^. The HVSC were cultured in advanced Dulbecco Modified Eagle's Medium (DMEM, Invitrogen) supplemented with 10% Fetal Bovine Serum (Greiner Bio-one), 1% penicillin/streptomycin (Lonza), 1% GlutaMax (Invitrogen). Only cells at passage 7 were used in this study. Myofibroblasts have been shown to be sensitive and respond to mechanical cues^44^,^45^. This makes this cell type interesting for our study.

### Micropost design and fabrication

The elastomeric microposts arrays were fabricated via standard photolithography processes, according to previous protocols^46^. Briefly, silicon wafers were patterned with an array of cylindrical pits. Afterwards, poly(dimethylsiloxane) (PDMS, Sylgard 184, Dow Corning), was poured over the silicon template, spincoated 30 seconds at 1000 rpm and cured at 110°C for 20 minutes to reach a Young's Modulus of 1.8 MPa. The micropost arrays were then peeled off the wafer. Our microposts were characterized by a radius r of 1 μm in case of circular cross section (circular microposts) and semi-major axis a of 1.50 μm and semi-minor axis b of 0.87 μm in case of elliptic cross section (elliptical microposts). The densely packed microposts (center to center distance, c.t.c. of 4 μm) formed arrays of 1.0 × 1.0 mm with spaces of 150 μm between them. The micropost lengths used in this study were (L) of 1, 3 and 6 μm. Together, we created a library of microposts for our modular setup. As the bending stiffness of each micropost is solely determined by its geometry, we modulated substrate stiffness by varying micropost length^47^,^48^. Finite element analysis was used to calculate the force–displacement relationship, leading to the micropost bending stiffnesses reported in Table 1 (Supplementary Fig. S2 online). Micropost arrays were bonded to glass coverslips (Menzel) or six-well plates (Uniflex Series Culture Plates, Flexcell FX 5000, Flexcell International, Hillsborough, NC, USA) using a corona discharger. To promote irreversible bonding, the sample was kept in oven at 60° overnight.

### Micro-contact printing on microposts

A flat PDMS stamp was cleaned and then incubated with 50 μg/ml rhodamine fibronectin (Cytoskeleton, Denver, CO, USA) in deionized water for 1 hour. The stamp was dried under sterile airflow and deposited gently on the micropost array, previously subjected to UV ozone treatment. A gentle pressure was applied to the stamp. The contact between the microposts and the stamp was ensured for at least 1 minute. Subsequently the substrates were sterlized in 70% ethanol and immersed in 0.4% Pluronics F127 (Sigma–Aldrich, St. Louis, MO) in PBS for 1 hour to prevent non-specific protein absorption to the non-functionalized surface of the PDMS microposts. Finally the substrates were rinsed with sterile MilliQ water and kept in PBS before use^46^.

### Loading protocol

Mechanical straining experiments were performed using FX-5000 Flexcell system (Flexcell Corp. (Mc-Keesport, PA)). Cells were seeded on the microposts at a density of 2500 cells/cm^2^ and were allowed to adhere overnight before the strain experiment (24 hours). The Uniflex membranes bound to the microposts were uniaxially and cyclically stretched (10%, 0.5 Hz) for 19 hours. The total duration of the experiment, from seeding to fixation, was 33 hours. Strain fields on the elastomeric microposts were characterized within the central region of the Flexcell membrane on which the microposts were bound by use of a Matlab-based (Mathworks Inc., Natick, MA) digital image correlation (DIC) code. A random pattern of fiducial markers was inked on the microposts bonded to the FlexCell membrane and images were recorded by mounted on a Zeiss stereomicroscope. By applying our uniaxial straining protocol (10%, 0.5 Hz, sine wave), results showed that in the area under consideration the strain field covers a range between 5% and 7%. Strains in the x direction (direction of applied strain) are 6.8% on average, and strains in y direction show a compression of 2%. Cells under static conditions grown for 33 hours on microposts bonded to glass coverslips were used as control.

### Immunofluorescence labelling

After culturing, HVSC were washed with PBS and fixed with 4% formaldehyde in PBS (Sigma-Aldrich) for 10 minutes at room temperature. Next, they were permeabilized with 0.1% Triton-X-100 (Merck) in PBS for 10 minutes and incubated with 3% bovine serum albumin (BSA) in PBS in order to block non-specific binding. Subsequently, samples were incubated with FITC-conjugated phalloidin (1:500, Phalloidin-Atto 488, Sigma) and DAPI (100 ng/ml, Fluka) for 1 hour at room temperature for immunofuorescence of F-actin and nucleus. Finally, the samples were rinsed in PBS and mounted onto glass slides using Mowiol.

### Microscopy and image analysis

For fluorescence visualization of actin stress fibers, cells were imaged using an inverted microscope (Zeiss Axiovert 200M, Zeiss, Gottingen, Germany), using 10 x/0.25 Ph1 e or 20 x/0.25 Ph1 objectives.

Imaging of basal fibers, actin cap fibers and nucleus was performed with an inverted confocal microscope (Zeiss LSM 510 META), using a C-Apochromat water-immersion objective (63×, NA = 1.2). The laser-scanning microscope was used according to the manufacturer's specification, using an argon laser at 488 nm (30 mW) for FITC-conjugated phalloidin and Chameleon (Chameleon Ultra II, Coherent, Santa Clara, CA) for DAPI at 760 nm. Z-series were generated by collecting a stack consisting of optical sections using a step size of 0.45-1.00 μm in the z-direction.

### Quantification of cell and nuclear orientation

Cell and nuclear orientation was measured with ImageJ. An ellipse was fitted to each cell outline and to the nucleus. The orientation angleθ, of the long axis of the ellipse with respect to the strain direction was measured.

### Quantification of stress fibers orientation

Fluorescence images of HVSC stained for actin stress fibers and confocal images of the basal fibers and actin cap were analyzed with Fiji (http://fiji.sc/Fiji)^49^. The orientation of actin stress fibers was calculated with respect to the straining direction for dynamic conditions or with respect to the x-axis of the image taken as a reference for static conditions. Fluorescence images of stress fibers were processed with Fiji before stress fiber tracking. Background subtraction and contrast enhancement were performed on the green channel images. Finally, directionality analysis for stress fiber orientation was conducted with Fiji using the Directionality plug-in (http://fiji.sc/wiki/index.php/Directionality) based on the FFT of each image by means of fitting a Gaussian to the FFT signal, measuring its peak position. The directionality algorithm takes into account only aligned elements in an entire image and is not sensitive to random elements. For each experiment, at least 40 cells were taken into consideration.

Analysis of the mean orientation of basal and actin cap fibers was calculated as follows. In the z-stacks, a rectangular region of interested was selected in order to fit the nucleus. The z-stack slices containing the basal and actin cap fibers were analyzed using the Directionality plug-in in Fiji.

### Data analysis

For each individual experiment the average fiber fraction for each angle was calculated. Subsequently, these fractions were fitted with a bimodal function. The fiber distributions were approximated by a bi-modal periodic normal probability distribution function using non linear least-square approximation algorithm^50^,^51^:
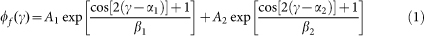
Hereby, *ϕ_f_*(*γ*) is the fiber fraction as a function of the fiber angle *γ*. Variables *α*_1_ and *α*_2_ are the two main fiber angles while *β*_1_ and *β*_2_ represent the dispersities of the two fiber distributions. For uniaxial cyclic straining, an angle of 90° is perpendicular to the strain direction. *A*_1_ and *A*_2_ are scaling factors for the total fiber fractions of the distributions. The quality of the bimodal approximation is represented by the R-squared value.

For the basal and actin cap fibers, analysis of the mean orientation was performed as follows. The main fiber directions (the one with least dispersity) for the basal and actin cap fibers were obtained from the bimodal fitting. The absolute values of these were averaged. Data for the basal and actin cap fibers mean orientation are expressed as mean ± SEM. Statistical significance of differences between the mean orientations of basal and actin cap of fibers was determined using one-way ANOVA followed by post-hoc Bonferroni multiple comparison tests. A p value of 0.05 was considered significant.

## Author Contributions

C.T., C.V.C.B. and F.P.T.B. designed the experiments; C.T. conducted the experiments, analyzed the data and wrote the manuscript text. All authors reviewed the manuscript.

## Supplementary Material

Supplementary Information Supplementary Information

## Figures and Tables

**Table 1:** Library of microposts used for the modular setup

micropost array type	radius/semi-axis (μm)	c.t.c. dist. (μm)	*L* (μm)	micropost bending stiffness *k* (nN/μm)
circular	*r* = 1	4	1	774
	*r* = 1	4	3	94
	*r* = 1	4	6	16
elliptical	*a* = 1.5	4	1	k(a) = 1252
	*b* = 0.87			k(b) = 895
	*a* = 1.5	4	3	k(a) = 215
	*b* = 0.87			k(b) = 98
	*a* = 1.5	4	6	k(a) = 41
	*b* = 0.87			k(b) = 16

In addition to the geometrical factors (radius/semi-axis (*r, a, b*), center to center distance (c.t.c.) and length (*L*)), also the corresponding micropost spring constants (*k*) are shown.
