# Supplementary material for: Competition between cap and basal actin fiber orientation in cells subjected to contact guidance and cyclic strain
Source: Sci Rep. 2015 Mar 4;5:8752. doi: 10.1038/srep08752 (PMC4348627; doi:10.1038/srep08752)
Supplement: Supplementary Information [file srep08752-s1.pdf]

Supplementary Information

Competition between cap and basal actin fiber orientation in cells subjected to contact guidance and cyclic strain

Authors: Chiara Tamiello, Carlijn C.V. Bouten, Frank P.T. Baaijens

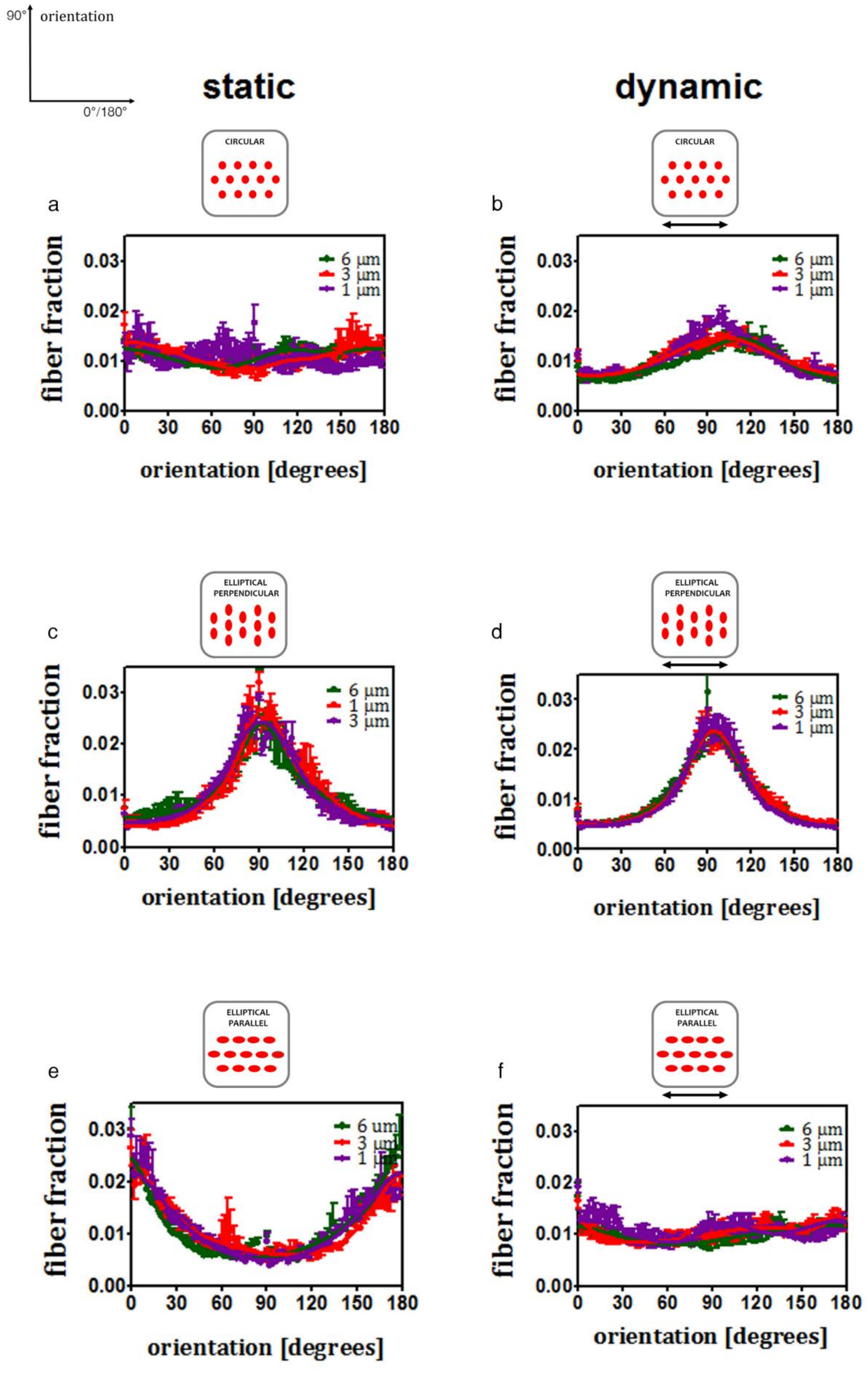
90°
orientation
0°/180°
static
dynamic
CIRCULAR
a
b
c
d
e
f
ELLIPTICAL PERPENDICULAR
ELLIPTICAL PARALLEL
6 µm
3 µm
1 µm
fiber fraction
orientation [degrees]

Figure S1. Stress fiber distribution is independent of micropost bending stiffness. Bimodal fits of the stress fiber orientation for each experimental condition. On each graph the stress fiber distributions for 1 μm, 3 μm and 6 μm microposts are reported. Data are represented by mean ± SEM. In static condition, 3 experiments were performed; in dynamic condition, 3 experiments were performed with 1 μm microposts, while 7 experiments were performed in the other experimental conditions.

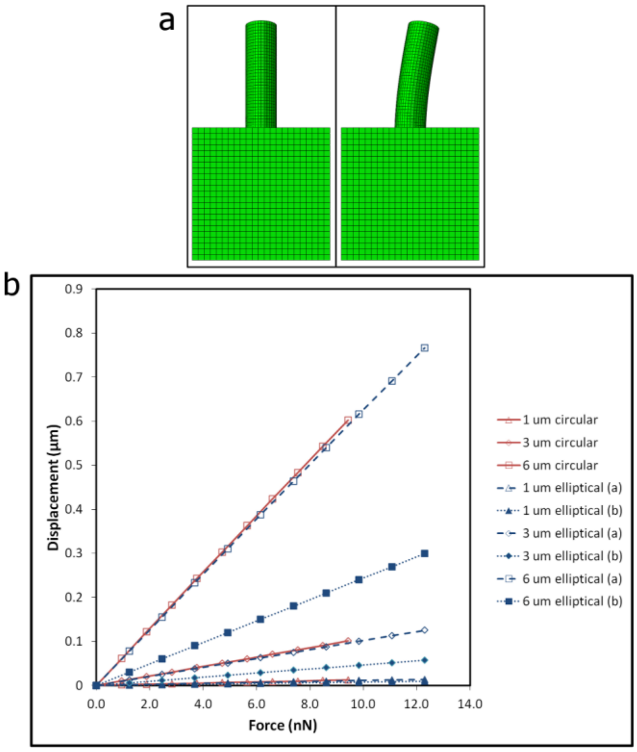

Figure S2. Micropost bending properties. (A) Finite element model of the micropost in its initial (left) and deformed shape (right). A thick layer of PDMS substrate underlying the micropost was taken under consideration in the FE simulations. (B) Plot of force vs. displacement as was calculated from the finite element model for each micropost type.
